# Engineering multi-specific antibodies against HIV-1

**DOI:** 10.1186/s12977-018-0439-9

**Published:** 2018-08-29

**Authors:** Neal N. Padte, Jian Yu, Yaoxing Huang, David D. Ho

**Affiliations:** Aaron Diamond AIDS Research Center, The Rockefeller University, 455 First Avenue, New York, NY 10016 USA

**Keywords:** HIV-1, Bispecific antibody, Trispecific antibody, Multi-specific antibody, Neutralizing antibody, Passive immunization

## Abstract

As increasing numbers of broadly neutralizing monoclonal antibodies (mAbs) against HIV-1 enter clinical trials, it is becoming evident that combinations of mAbs are necessary to block infection by the diverse array of globally circulating HIV-1 strains and to limit the emergence of resistant viruses. Multi-specific antibodies, in which two or more HIV-1 entry-targeting moieties are engineered into a single molecule, have expanded rapidly in recent years and offer an attractive solution that can improve neutralization breadth and erect a higher barrier against viral resistance. In some unique cases, multi-specific HIV-1 antibodies have demonstrated vastly improved antiviral potency due to increased avidity or enhanced spatiotemporal functional activity. This review will describe the recent advancements in the HIV-1 field in engineering monoclonal, bispecific and trispecific antibodies with enhanced breadth and potency against HIV-1. A case study will also be presented as an example of the developmental challenges these multi-specific antibodies may face on their path to the clinic. The tremendous potential of multi-specific antibodies against the HIV-1 epidemic is readily evident. Creativity in their discovery and engineering, and acumen during their development, will be the true determinant of their success in reducing HIV-1 infection and disease.

## Background

The past decade has introduced a new generation of potent and broad neutralizing monoclonal antibodies (mAbs) against HIV-1 [1–10], several of which have entered the clinic recently [11–17]. This resurgence of promising HIV-1 mAbs has energized the field of passive immunization and propelled the testing of existing mAbs as treatment, particularly because of their long half-lives as compared to existing oral antiretroviral options. The high degree of HIV-1 envelope (Env) diversity, however, requires further improvements to these mAbs to better ensure their clinical utility. For example, viral resistance can rapidly evade antiviral pressure from a single mAb treatment [11, 12, 14, 18, 19], and a large fraction of circulating HIV-1 already exhibit pre-existing resistance to many of the antibodies currently in development [20–22].

HIV-1 mAbs directed to more conserved components of the viral entry process, such as ibalizumab, which binds to the CD4 receptor on T-cells [23], and PRO140, which binds to the CCR5 co-receptor [24], broadly neutralize a greater fraction of circulating HIV-1 than Env-targeting mAbs [20, 25]. Indeed, ibalizumab (Trogarzo^®^) has recently become the first mAb against HIV-1 to receive FDA approval and is currently indicated for use as salvage therapy in patients whose viruses are resistant to multiple existing antiretroviral drugs [26, 27]. PRO140 is currently in a Phase 2b/3 pivotal trial in heavily treatment-experienced HIV-1 patients [28]. However, these promising antibodies must be used in combination with other antiretroviral agents to limit emerging viral resistance. While the newer generation of Env-targeting mAbs that have recently entered Phase 1 trials are more potent and broad than earlier generations of HIV-1 Env-targeting mAbs, they still face these same issues of viral resistance unless they can be administered in combinations, and this costly undertaking could limit their practical feasibility, particularly in the setting of HIV-1 prevention in under-resourced nations [29]. Engineering antibodies for greater HIV-1 neutralization and breadth, particular by the creation of bispecific and trispecific antibodies, and for improved in vitro stability and in vivo pharmacokinetics, has the potential to drastically reduce the amount of antibody required for efficacy in humans, and may put the goal of an efficacious HIV-1 prevention and therapeutic antibody strategy within reach.

## Engineering mAbs to improve potency and breadth against HIV-1

One strategy to improve HIV-1 mAbs is to use structure-guided design to develop rationally engineered antibody variants with improved antiviral properties. Many of the engineering principles applied to these HIV-1 mAbs were also incorporated into the investigational studies to engineer multi-specific antibodies reviewed in this article, and therefore a short summary of these structure-guided engineering approaches for HIV-1 mAbs will be reviewed first.

### Engineering CD4 binding site mAbs

The HIV-1 CD4 binding site antibody NIH45–46 was identified as a more potent clonal variant of VRC01 [6, 10]. Structural studies determined that NIH45–46 lacked a critical interaction to a hydrophobic pocket between the gp120 bridging sheet and outer domain that is typically occupied by a phenylalanine on CD4, and it was reasoned that a hydrophobic residue at position 54 on NIH45–46 could improve its interaction with gp120. After engineering one of a series of hydrophobic residues at this position 54, the variant NIH45–46^G54W^ was found to increase contact with the gp120 bridging sheet and improved its neutralization potency by tenfold [30].

VRC07, another somatic variant of VRC01, was engineered with improved binding to the HIV-1 CD4 binding site by incorporating a histidine mutation at the G54 position of this antibody (the same position as that mutated in NIH45–46^G54W^). VRC07 was also engineered with several mutations in its light chain to increase solubility and to remove a potential N-linked glycosylation site, which together resulted in a 7.9-fold enhancement in potency as compared to VRC01 and with reduced autoreactivity as compared to NIH45–46^G54W^ [5]. A variant of VRC07-523 engineered to have a longer half-life in vivo (VRC07-523-LS) demonstrated protective efficacy at one-fifth of the dose of VRC01-LS in a non-human primate model, and is currently in Phase 1 clinical evaluation [16].

### Engineering MPER binding site mAbs

A similar approach to improve antibody solubility and potency was taken for the gp41 membrane proximal external region (MPER) binding antibody, 10E8 [3]. 10E8 was identified from an HIV-1 infected individual and is one of the broadest antibodies reported to date, neutralizing > 95% of circulating HIV-1 strains. However, 10E8 is naturally prone to aggregation, which limited its clinical manufacturability potential. By identifying somatic variants of 10E8 with inherently better solubility, and then using structural data to mutate a hydrophobic patch distal from the binding site of this antibody, a significantly more soluble variant of 10E8 was obtained [31]. Because germline variants often exhibit reduced potency compared to their affinity matured antibody counterparts, residues from 10E8 critical for binding to MPER were then grafted onto this more soluble antibody. The new 10E8 variants retained the improved solubility but now also exhibited potency similar to the originally identified 10E8. The top variants, 10E8v4 and 10E8v5, exhibited improved pharmacokinetic profiles in mice and rhesus macaques as compared to 10E8, and 10E8v5 has been advanced for clinical evaluation [32]. An additional 10E8v4 variant, known as 10E8v4-5R + 100cF, was recently reported to improve the potency of 10E8v4 by an additional ~ 10-fold using a surface-matrix screening approach [33].

### Engineering a CD4-targeting mAb

In addition to engineering antibodies for improved solubility and potency against HIV-1, improved breadth of neutralization against circulating HIV-1 strains has also been demonstrated, which has the potential to erect a higher genetic barrier to viral resistance. The aforementioned CD4-targeting antibody, ibalizumab, already demonstrated favorable potency and breadth against circulating HIV-1 strains [20]. It neutralized 92% of viruses tested in vitro as assessed by ≥ 50% neutralization, but only neutralized 66% of viruses when assessed as ≥ 80% inhibition. This indicated that a significant fraction of circulating viruses may be able to escape complete neutralization. These studies revealed a strong correlation between HIV-1 resistance to ibalizumab and a loss of a V5 glycan on the viral envelope. In a separate study in HIV-1 infected patients in which ibalizumab monotherapy was added to failing drug regimens, a transient decrease in viral load was followed by evolution of resistant HIV-1 variants with a similar loss of a V5 glycosylation site [19]. Taken together with epitope mapping and X-ray crystallography structural studies used to define the ibalizumab-CD4 binding interface [34, 35], it was hypothesized that the loss of the HIV-1 V5 glycan provided the viral envelope more flexibility to circumvent the steric hindrance induced by ibalizumab. To address this deficiency in ibalizumab, a panel of variants was engineered with glycans added to the ibalizumab light chain at positions predicted to sterically fill the empty space created by the loss of V5 glycan in the resistant viruses [36]. These modified glycan variants were able to neutralize HIV-1 strains previously resistant to ibalizumab, and the top variant, known as LM52, neutralized 100% of circulating HIV-1 strains tested as assessed by ≥ 80% neutralization, and at a potency ~ 5- to 10-fold better than wild-type ibalizumab. LM52 is currently in preclinical development in preparation for clinical evaluation [37].

The examples presented above demonstrate how structure-guided approaches and rational design, in combination with germline antibody identification, can improve the potency, breadth and solubility of multiple antibodies against HIV-1, and several of these are currently in preclinical or clinical development. However, even with these improvements, the dynamics of HIV-1 viral replication and the rapid mutation rate of HIV-1 require these antibodies be used in combinations in order to limit the emergence of resistant viruses in a treatment setting and in order to block infection by a diverse range of circulating subtypes in a prevention setting. While such combinations of antibodies are currently being explored [22], the high cost of development and delivery of these biologic combinations has the potential to limit their widespread use, necessitating alternative solutions.

## Engineering multi-specific antibodies to improve breadth against HIV-1

The idea that multi-specific antibodies could improve upon the functional activities of single mAbs or combinations of mAbs originated in the cancer therapy field in the mid-1980s, primarily as a way to direct effector cells toward tumor cells [38–40]. As a result, the majority of bispecific antibodies currently under clinical evaluation today are for the treatment of various cancers [41]. The need for multi-specific antibodies for HIV-1 prevention and treatment, however, is readily evident. Multiple HIV-1 targeting epitopes can be incorporated into one antibody-like molecule, allowing for increased neutralization breadth against diverse HIV-1 strains and thereby also erecting a higher genetic barrier for viral resistance. Additionally, the large array of multi-specific antibody formats currently available [42] allow the tailoring of any particular combination of HIV-1 targeting antibody moieties by a number of structural properties such as size, distance, and valency in order to meet the requirements of viral inhibition.

### Engineering bispecific antibodies with improved breadth

**Fig. 1:**
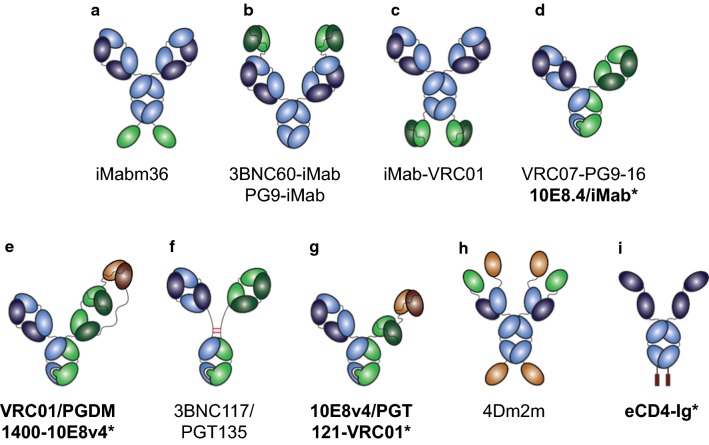
Multi-specific antibody formats engineered for the prevention and treatment of HIV-1. **a** IgG-Fv fusion, **b**, **c** IgG-scFv, **d** CrossMAb, **e** KiH-CODV-IgG, **f** IgG3C-, **g** KiH + tandem scFvs, **h** tetravalent + bivalent Fc-fusion, **i** Fc-fusion peptide. Representative multi-specific antibodies are listed under their respective schematic. *Means currently in clinical development

One example of a bispecific antibody that can enhance neutralization breadth is iMabm36 [43], which inhibits HIV-1 entry by targeting CD4, via ibalizumab (iMab), and the gp120 co-receptor binding site, via the antibody domain m36. This bispecific antibody is generated by genetically linking m36 to the C-terminus of the ibalizumab heavy chain (Fig. 1a). As stated earlier, ibalizumab neutralizes 66% of viruses when assessed as ≥ 80% inhibition, indicating a significant fraction of circulating viruses may escape complete neutralization by ibalizumab. In contrast, the bispecific antibody iMabm36 neutralized 87% of viruses as defined by ≥ 80% inhibition, indicating a substantial improvement in neutralization breadth. This is attributed to the presence of two distinct HIV-1 entry inhibiting antibody domains within the same molecule. Improved antiviral activity was dependent on both the CD4-binding activity of the iMab component as well as the gp120 coreceptor-binding activity of the m36 component, as knocking out the activity of either of these components within the iMab36 molecule greatly reduced its antiviral activity. The linker length between the m36 antibody domain and the C-terminus of the iMab heavy chain also affected the antiviral activity of the bispecific antibody, suggesting that the flexibility and position of the fused domains relative to one another are also important for the functional activity of iMabm36.

In a separate line of study, a panel of bispecific antibodies was engineered in which one of several gp120-targeting single-chain variable fragments (scFv) was fused to the N-terminus or C-terminus of the ibalizumab heavy chain (Fig. 1b, c) [44]. A number of variations of this format were also engineered, including those which inverted the orientation of variable domains within the scFv (for example, V_H_ followed by V_L_, or V_L_ followed by V_H_), and those which varied the linker lengths between the V_H_ and V_L_ domains within each scFv or between the scFv domains and the ibalizumab heavy chain. Interestingly, the binding and neutralization activity of each of these bispecific antibody variants varied widely, and the most optimal format in terms of V_H_ and V_L_ orientation and linker lengths differed depending on which HIV-1 Env-targeting scFv was fused to ibalizumab. Therefore, identifying an optimal bispecific antibody format and design, even within the context of structure-guided rational design of HIV-1 antibody-epitope pairings, is still an empirical process.

Ibalizumab fused to gp120 CD4 binding site antibodies, such as VRC01, NIH45–46^G54W^, or 3BNC60, neutralized > 99% of circulating HIV-1 strains tested, as assessed by ≥ 50% neutralization, and with a geometric mean IC_50_ ranging from 0.025 to 0.031 µg/mL. These bispecific antibodies also neutralized > 97% of strains tested, as assessed by ≥ 80% inhibition, with a geometric mean IC_80_ ranging from 0.076 to 0.092 µg/mL. This significant enhancement in neutralization breadth when ibalizumab was fused to each of these gp120 CD4 binding site antibodies indicates that these sets of parental antibody pairings could prove optimal in neutralizing a diverse sequence of circulating HIV-1 strains. Indeed, as mentioned earlier, a strong correlation was observed between HIV-1 resistance to ibalizumab and a loss of a V5 glycan on the viral envelope [20] and, in contrast, resistance to VRC01 involves the presence of bulky V5 residues [45]. Therefore, by combining two antibodies with complimentary resistance profiles into a single bispecific antibody, tremendous enhancements in neutralization breadth at or close to 100% can be achieved.

The CrossMAb format for engineering bispecific antibodies, originally developed by Roche, has also been utilized for HIV-1 antibody development in recent years. The CrossMAb format allows for correct assembly of two heavy chains and two light chains from different antibodies into one bispecific antibody molecule that resembles a typical monoclonal antibody in terms of mass and architecture, and with no artificial linkers required (Fig. 1d) [46]. This is achieved by combining the knob-into-hole technology, which enables heterodimerization of two different heavy chains, and the light chain crossover technology, which ensures correct association of each of the light chains with their cognate heavy chains.

In one study, CrossMAb antibodies targeting four major HIV-1 Env epitopes known to be important for HIV-1 neutralization, the CD4 binding site, V3 glycan, V1V2, and MPER regions, were engineered [47]. These HIV-1 CrossMAb bispecific antibodies neutralized 95–97% of circulating HIV-1 strains tested, and the most promising candidate from this study, VRC07-PG9-16, neutralized the panel of viruses with a median IC_50_ of 0.055 µg/mL. This represented an improvement in neutralization breadth and coverage over the single parental mAbs from which VRC07-PG9-16 was derived, and was similar in breadth and potency to the co-administration of the two parental mAbs, which was not the case for all of the bispecific antibodies engineered and evaluated.

### Engineering trispecific antibodies with improved breadth

It is now well known that the highly dynamic nature of HIV-1 replication in vivo demands treating HIV-1 with three antiretroviral agents simultaneously since viral escape against any single agent is an inevitable consequence of the large number of HIV-1 mutants generated per day within an infected person [48–50]. With this in mind, the continuous evolution of HIV-1 Env during the course of infection also attests to the exceptional selective pressure exerted by naturally elicited virus-specific antibodies [51]. Therefore, trispecific antibodies with the potential to inhibit viral entry with three distinct HIV-1 Env-targeting antibody moieties was of interest. In one study, the trispecific antibodies VRC01/PGDM1400-10E8v4 and N6/PGDM1400-10E8v4 were engineered using a knob-in hole (KiH) heterodimerization technology [52] and a cross-over dual variable immunoglobulin G (CODV-Ig) technology to ensure affinity of each variable region was maintained [53] in order to target the HIV-1 envelope CD4 binding site, MPER and V2 glycan site (Fig. 1e) [54]. Multiple combinations of broadly neutralizing parental antibodies and formats were tested before downselecting VRC01/PGDM1400-10E8v4 and N6/PGDM1400-10E8v4, which demonstrated 98% and > 99% breadth, respectively, as defined by ≥ 50% neutralization. Surface plasmon resonance confirmed that each of the three antibody-targeting domains within VRC01/PGDM1400-10E8v4 had comparable affinities for its HIV-1 Env antigens relative to its parental Fab counterparts. VRC01/PGDM1400-10E8v4 also provided 100% protection to nonhuman primates challenged mucosally with a mixture of two SHIVs, SHIV 325C and SHIV BaLP4, which each had varying sensitivities to two of the parental mAb counterparts of VRC01/PGDM1400-10E8v4, while only 62% and 75% of nonhuman primates administered VRC01 or PGDM1400, respectively, were protected in this model. Therefore, the improvement in neutralization breadth observed by VRC01/PGDM1400-10E8v4 in vitro translated to an improved breadth of protection against SHIV in vivo.

While the bispecific and trispecific antibodies discussed above enhanced HIV-1 neutralization breadth relative to their parental mAb counterparts, they were limited in their ability to enhance potency relative to the parental mAbs provided individually or in combination. This is thought to be due, in part, to the low spike density of gp160 trimers on the surface of HIV-1 [55–57], which may limit the ability of these bispecific and trispecific antibodies to bind to the HIV-1 envelope bivalently (or trivalently in the case of a trispecific antibody) through inter-spike crosslinking. The gp160 trimer spike structure itself may also limit the ability of these multi-specific antibodies to achieve intra-spike crosslinking [55, 56]. While antibodies elicited naturally during HIV-1 infection also typically interact monovalently with the HIV-1 gp160 trimer spike, polyreactive antibodies have been proposed to be positively selected and retained during affinity maturation and can increase their overall apparent affinity for HIV-1 Env through heteroligation [58]. The VRC07-PG9-16 CrossMAb discussed earlier can achieve a potency similar to, but not better than, the most potent of its parental mAbs against any particular virus, and this is thought to be due to an inability of VRC07-PG9-16 to simultaneously bind both of its epitopes on the HIV-1 Env trimer [47]. If multivalent binding of these bispecific or trispecific antibodies was possible, one could imagine that a significant enhancement in antiviral potency could be gained in addition to enhanced breadth.

## Engineering multi-specific antibodies to improve breadth and potency against HIV-1

One study has investigated the importance of this potential for enhanced HIV-1 neutralization by inter- and intra-spike binding by using DNA as a “molecular ruler” that has a HIV-1 Env binding antibody domain conjugated to each end [59]. By increasing or decreasing the number of basepairs (bp) between two Fabs of either 3BNC60 [6] or VRC01 [10], homo-dimer Fabs with different lengths of “reach” were used to probe the distance needed to achieve avidity as opposed to single arm Fab binding. These studies revealed that a length of ~ 60 bp resulted in ~ 100-fold increased potency for either 3BNC60 or VRC01 homo-diFabs against the specific HIV-1 strain tested, likely due to bivalent binding to two CD4 binding sites within a single gp120 trimer. Hetero-diFabs also exhibited enhanced potency as compared to combinations of their monoclonal antibody counterparts. For example, a PG16-3BNC60 diFab, targeting both V1V2 and the CD4 binding site in a single gp120 trimer, enhanced neutralization potency by ~ 100-fold when a 50 bp double-stranded (ds) DNA bridge was used to separate these two Fabs. The 50–60 bp ds DNA bridges in these molecules represent a reach distance of ~ 17–21 nm between the two Fabs in a single molecule, which is longer than the ~ 12–15 nm reach of two Fab arms in a typical IgG molecule [55]. While the molecular flexibility and dynamics that may be associated with an antibody binding to either the open or closed HIV-1 Envelope trimer may somewhat alter these distances in a case-dependent manner, it is generally thought that the reach between the two Fab arms in a HIV-1 multi-specific antibody would need to be larger than that within a typical IgG in order to capture the benefits of avidity and multivalent binding. These DNA diFab constructs provide an elegant method to investigate the science underlying antibody avidity to HIV-1 Env, but are not readily translatable to product development and clinical use.

All of the bispecific antibodies discussed until now have utilized an IgG1 or IgG4 subtype, based on their intended mechanism of action. Another subclass, IgG3, possesses a relatively longer and more flexible hinge domain region [60, 61], which may allow for the greater “reach” needed to achieve bivalent binding of a bispecific antibody against HIV-1 Env. To test this, a small panel of CrossMAb format HIV-1 bispecific antibodies were generated in which the typical IgG1 hinge domain was replaced with a longer and more flexible IgG3 hinge-like region called IgG3C- (Fig. 1f) [62]. One of these IgG3C- hinge variants that targeted the CD4 binding site and V3 region of the HIV-1 envelope, 3BNC117/PGT135, exhibited both superior breadth (93% as defined by 50% inhibition and 89.1% as defined by 80% inhibition) and superior potency (IC_50_ geometric mean of 0.036 µg/mL and IC_80_ geometric mean of 0.159 µg/mL) relative to its single parental mAbs or the predicted combination of both parental mAbs. Variants in which the IgG3C- hinge length of 3BNC117/PGT135 were decreased resulted in decreased neutralization activity. Combined with structural data modeling 3BNC117 and PGT135 Fabs complexed with the Env trimer, this suggests that the IgG3C- hinge variant of 3BNC117/PGT135 may allow for bivalent binding, enhanced avidity, and ultimately greater potency relative to its parental mAb counterparts. No differences in the pharmacokinetic profile of this bispecific antibody were observed in mice in comparison to typical mAbs, and a 1.5 log_10_ decrease in viral load was observed in a humanized mouse model for HIV-1 treatment. In comparison, treatment with a mixture of the 3BNC117 and PGT135 parental mAbs yielded very little change in viral loads.

Another study reported the engineering of trispecific antibodies in order to increase “reach” and improve HIV-1 neutralization breadth and potency. Using scFv domains connected in tandem with flexible linkers, different formats of scFv domains targeting the HIV-1 CD4 binding site, V3, and MPER regions were engineered and characterized for their ability to improve antiviral activity and HIV-1 Env binding avidity (Fig. 1g). From these studies, 10E8v4/PGT121-VRC01 emerged as the most promising trispecific antibody candidate, exhibiting 99.5% breadth, as defined by 50% inhibition, an IC_50_ geometric mean of 0.069, and an IC_80_ geometric mean of 0.298 µg/mL [63]. Biolayer interferometry was used to confirm that all three scFv domains in this trispecific antibody could bind to their cognate HIV-1 Env epitopes, and it is suggested that the four-fold enhancement in potency of 10E8v4/PGT121-VRC01 relative to its parental mAbs is due to the cooperative effect of binding to at least two epitopes simultaneously on the HIV-1 Env trimer.

In addition to bispecific and trispecific antibody formats, smaller Fc fusion proteins have also been engineered with the goal of improving potency by enabling bispecific avidity. 4Dm2m is comprised of a single domain of soluble CD4, known as mD1.22, fused to the N- and C-termini of a human IgG1 heavy chain constant region, and an antibody domain targeting the coreceptor binding site on gp120, known as m36.4, fused to the N-terminus of the human antibody light chain constant region via a glycine-serine linker (Fig. 1h) [64, 65]. This bispecific multivalent fusion protein neutralized all HIV-1 isolates tested with a potency about 10-fold higher than the CD4 binding site antibody, VRC01. The authors reasoned that the improvement in potency between 4Dm2m and a variant with m36.4 only at the N-termini, known as 2Dm2m, was due to bivalent binding of both the head and tail m36.4 antibody domains in 4Dm2m and the relative close proximity of the CD4 binding site and coreceptor binding site on gp120.

eCD4-Ig is a fusion of CD4-Ig, which itself is comprised of CD4 domains 1 and 2 fused to Fc, and a small CCR5-mimetic sulfopeptide (Fig. 1i) [66]. eCD4-Ig neutralized 100% of a diverse panel of circulating HIV-1 strains, and could also neutralize HIV-2 strains, and this outstanding antiviral breadth is thought to be due to the relatively well conserved nature of the CD4 binding site and CCR5 coreceptor binding site epitopes on HIV-1 Env. A structural model of eCD4-Ig bound to the HIV-1 Env trimer predicts that both the CD4-Ig and CCR5-mimetic sulfopeptide bind avidly and cooperatively to HIV-1. This would support the high potency of eCD4-Ig, neutralizing a panel of HIV-1 with a geometric mean of < 0.05 µg/mL, as defined by 50% inhibition. eCD4-Ig variants neutralized each particular HIV-1 strain tested with a potency 10- to > 200-fold better than CD4-Ig alone. A rhesus version of one of the bispecific fusion variants, known as rh-eCD4-IgG2^I39N,mim2^, was cloned into an adeno-associated virus serotype 2 (AAV2) vector and, when co-administered with a separate single-stranded AAV vector expressing rhesus tyrosine-protein sulfotransferase to promote rh-eCD4-Ig sulfation, provided 100% protection against repeated SHIV-AD8 challenges. Recently, an improved variant of eCD4-Ig that utilized mD1.22, the stabilized form of CD4 domain 1 discussed earlier, was shown to improve the potency of this bispecific fusion peptide by another 9-fold while maintaining satisfactory production efficiency [67].

The antibodies discussed above demonstrate the principle that engineering multi-specific antibodies against HIV-1 for increased avidity can increase their antiviral potency and breadth. However, the large divergence in HIV-1 Envs and their relative dynamic nature pose a challenge to identifying multi-specific molecules with sufficient reach to consistently interact with target epitopes across diverse HIV-1 strains. Another approach to increase avidity and potency is to exploit the dynamic nature of HIV-1 Env to identify at least two antiviral targets in the overall viral entry process. By investigating the spatiotemporal process of HIV-1 entry, it was plausible that new combinations of bispecific antibody targets could be discovered that were not exclusive to targeting HIV-1 Env.

PG9-iMab and PG16-iMab, comprised of the scFv of the V1V2-targeting PG9 or PG16 mAbs fused to the CD4-targeting mAb ibalizumab, are two such examples (Fig. 1b) [68]. PG9-iMab and PG16-iMab both exhibited impressive breadth and potency, neutralizing 100% of viruses tested, as defined by 50% inhibition. When defined as 80% inhibition, PG9-iMab still neutralized 100% of viruses while PG16-iMab neutralized 98% of viruses. The enhancement in potency was also remarkable, with PG9-iMab exhibiting an IC_50_ geometric mean of 0.004 μg/mL and an IC_80_ geometric mean of 0.017 μg/mL, and PG16-iMab exhibiting an IC_50_ geometric mean of 0.003 μg/mL and an IC_80_ geometric mean of 0.015 μg/mL. The enhancement in potency was > 20-fold compared to the parental mAb ibalizumab and > 100-fold compared to the parental mAb PG9 or PG16, and far better than a co-mixture of the two parental mAbs together. Importantly, the ability of PG9-iMab to bind both CD4 on the T cell and V1V2 on HIV-1 Env did not result in any obviously detrimental form of crosslinking that could enhance viral activity in the TZM-bl and PBMC neutralization assays evaluated, but rather only potently and broadly inhibited viral activity. In some cases, the potencies of these bispecific antibodies were improved up to four-logs compared to their parental mAb counterparts. Mechanistic studies determined that the enhanced potency of PG9-iMab required anchoring of this bispecific antibody to CD4 via its ibalizumab component. Additional modeling studies suggest that this anchoring to CD4 positions the PG9 scFv component of PG9-iMab so that it can more easily interact with the V1V2 epitope on the Env of the incoming viral particle. In effect, this increases the local concentration of PG9 scFv precisely at the site where it can exert its antiviral activity.

Interestingly, the enhancement in potency observed with PG9-iMab in this scFv bispecific format was not replicated with other scFv bispecific combinations such as VRC01-iMab, 3BNC60-iMab or 45-46-iMab, which target CD4 via ibalizumab and the HIV-1 Env CD4 binding site via VRC01, 3BNC60, or NIH45–46 scFv domains [44]. However, an enhancement in potency was observed with the CD4- and HIV-1 Env V3-targeting PGT123-iMab, PGT128-iMab and 10-1074-iMab, approaching the level of potency observed with PG9-iMab or PG16-iMab. This suggests that, similar to a preferred accessibility to the HIV-1 Env V1V2 epitope when PG9-iMab and PG16-iMab are anchored to CD4, the HIV-1 Env V3 epitope may be similarly accessible when PGT123-iMab, PGT128-iMab or 10-1074-iMab are bound to CD4 [44].

While several scFv-format bispecific antibodies are currently in development, several properties inherent to this bispecific antibody format must be addressed before they can be advanced into the clinic. For example, the linker fusing the V_H_ and V_L_ domains of the scFv moiety, and the linker fusing the scFv moiety to either an IgG-like molecule or another scFv moiety, must be sufficiently flexible so as not to impair the normal folding and function of the binding domains within the bispecific antibody, must be sufficiently stable so as to avoid cleavage and subsequent separation of the antibody binding domains during manufacture or in vivo, and must be sufficiently soluble so as to avoid potential aggregation. The ideal linker length and orientation of the V_H_ and V_L_ domains within the scFv moiety may also vary depending on the biophysical properties and mechanism of action of the particular bispecific antibody. All of these properties vary from molecule to molecule, and must be empirically investigated and optimized during the development process. Finally, the unnatural architecture of many scFv-format bispecific antibodies, which may deviate significantly from typical IgG antibodies, or their associated linkers, may create neoantigens or expose cryptic epitopes that may lead to immunogenicity in vivo [69]. While several in silico or in vitro methods may be able to identify potential hotspots of antibody immunogenicity, host immune responses cannot be predicted solely by these methods [70], and the ultimate test of antibody immunogenicity is by clinical study [71].

As discussed earlier, the CrossMAb bispecific antibody format retains more of a native IgG-like structure and avoids the need for foreign linker sequences [46], which may obviate some of the development challenges associated with scFv bispecific antibodies. However, the native-like structure of CrossMAbs may also restrict the “reach,” and consequently the avidity, of two HIV-1 Env epitope binding variable domains when incorporated into this format [47]. Directing bispecific antibodies to host cell receptors with one of the CrossMAb arms, however, while targeting the other CrossMAb arm to the HIV-1 envelope, could take advantage of the dynamic nature of the HIV-1 entry process and allow for avidity by binding two HIV-1 entry targets simultaneously, similar to what was achieved with the PG9-iMab scFv format bispecific antibody. One study constructed and characterized a panel of 20 CrossMAb bispecific antibodies in which one arm inhibited HIV-1 by targeting the CD4 receptor or the CCR5 coreceptor via ibalizumab (iMab) or PRO140 (P140) [23, 24], and the other arm targeted the HIV-1 envelope MPER, CD4 binding site, V3 region, V1V2 region, or gp41–gp120 interface via 10E8, 3BNC117, PGT128, PGT145 or PGT151 [1, 3, 6, 8], and an optimal combination was identified which yielded exquisite antiviral potency and breadth [25]. The HIV-1 CrossMAbs 10E8/iMab and 10E8/P140 exhibited IC_50_ geometric means of 0.002 μg/mL and 0.001 μg/mL, respectively, and neutralization breadth (as assessed by ≥ 50% neutralization) of 100% and 99%, respectively. This represented a synergistic enhancement in potency hundreds of fold greater than those of its parental mAbs, and represented some of the most potent bispecific antibodies against HIV-1 identified to date. Interestingly, a CrossMAb comprised of a CD4-targeting ibalizumab arm and a V1V2-targeting PGT145 arm did not enhance antiviral potency, even though the CD4/V1V2-targeting PG9-iMab yielded a synergistic enhancement in potency in a scFv bispecific format [68]. Based on structural modeling data of the PG9-iMab scFv bispecific antibody discussed earlier, it is possible that the PG9 moiety may not be positioned at the right angle or length to neutralize HIV-1 Env when it is bound to CD4 or CCR5 in a CrossMAb format. Both 10E8/iMab and 10E8/P140 CrossMAbs, similar to the PG9-iMab scFv bispecific antibody, exerted their impressive antiviral activity by anchoring 10E8 near the two receptors HIV-1 utilizes, CD4 and CCR5, essentially placing 10E8 at precisely the right place and right time to bind HIV-1 Env MPER and potently neutralize an incoming viral particle. Indeed, if either the 10E8 or ibalizumab arm in 10E8/iMab (or the 10E8 or PRO140 arm in 10E8/P140) was engineered for reduced binding, the antiviral activity of the mutant bispecific was only as good as the mAb represented by the remaining intact arm within each of the bispecific CrossMAbs. After several rounds of antibody engineering to identify variants of these HIV-1 CrossMAbs with improved physicochemical homogeneity, an optimized variant known as 10E8_V2.0_/iMab (renamed 10E8.2/iMab) emerged with improved physicochemical properties, two-fold enhancement in bioavailability, and further improvement in antiviral potency compared to its predecessor (IC_50_ geometric mean of 0.002 μg/mL and IC_80_ geometric mean of 0.006 μg/mL). 10E8.2/iMab also demonstrated impressive antiviral activity in vivo, reducing viral load in HIV-1-infected humanized mice by 1.7 log_10_ and providing 100% protection against multiple systemic challenges with the tier-2 R5 virus, JR-CSF. Utilizing in vitro neutralization data for 10E8.2/iMab and other HIV-1 mAbs against subtype A, C, and D pseudoviruses, a model of neutralization potency and breadth for single and two mAb combinations predicted that this single bispecific molecule, 10E8.2/iMab, could provide broader and more potent protection across subtypes as compared to all two mAb combinations evaluated [22].

## Bispecific antibody development challenges

The impressive potency, breadth and higher barrier against emerging resistant viruses that can be achieved with HIV-1 bispecific or trispecific antibodies warrants their further investigation. In addition, the ability to capture this impressive antiviral activity in a single multi-specific molecule, as opposed to combinations of multiple mAbs, makes the development of HIV-1 bispecific and trispecific antibodies an attractive path commercially. One HIV-1 multi-specific molecule could achieve the same or better antiviral activity as combinations of multiple mAbs, but the manufacturing, storage, transport and administration costs remain similar to that of a single agent.

However, while the manufacturing process for typical mAbs is relatively mature and established, unexpected manufacturing challenges unique to each bispecific or trispecific antibody format must be overcome in order to make development of these multi-specific molecules a feasible strategy for HIV-1 treatment or prevention. Some of the challenges of scFv format bispecific antibodies were discussed earlier, such as the potential for linker instability, aggregation propensity and potential immunogenicity in vivo due to the difference in architecture between these bispecific molecules and typical IgG antibodies. Additionally, the non-native structure of this bispecific antibody format could result in a poor pharmacokinetic profile in vivo. Other bispecific formats, such as the CrossMAb format, avoid the use of linkers and maintain a more natural IgG antibody architecture while still achieving bispecificity as asymmetric IgG heterodimers. However, because two distinct heavy chains and two distinct light chains are required to produce the desired product, homodimer byproducts or light chain mispairings may arise and must be overcome.

Downstream processes may also possess unique challenges. While typical mAbs are purified using a Protein A resin that binds to the Fc region of the mAb, and then additional purification polishing steps are performed as necessary, bispecific antibodies that utilize asymmetry, such as the CrossMAb format, cannot be distinguished from homodimer impurities since the Fc regions of both the target heterodimer product and the impurity consisting of homodimers would interact equally well with Protein A. These bispecific formats must exploit asymmetry to their advantage in their purification processes as well, for example, by using a kappa light chain with one arm of the intact molecule and a lambda light chain with the other arm of the intact molecule so that successive rounds of purification that capture each of the light chain arms sequentially would allow for purification of the intact molecule [42]. Other purification tools that can take advantage of asymmetry could also be employed, such as engineering each bispecific antibody arm with sufficient differences in isolectric points so that sequential purification by anion exchange and cation exchange chromatographies would result in purified heterodimers. Additionally, the combination of difficult upstream production procedures for certain complex bispecific antibody formats and multiple downstream purification steps may result in lower final product yields for bispecific antibodies as compared to typical mAbs.

Nonetheless, the tremendous therapeutic potential of HIV-1 bispecific and trispecific antibodies, with evidence of synergistically enhancing antiviral activity by several logs and the potential for drastically lower production costs by containing the therapeutic to a singular molecular entity, necessitate strategies be developed to overcome these challenges. By embarking on a scientifically rigorous approach towards developability and manufacturability that combines elements of quality by design with a deep mechanistic understanding of the specific therapeutic, promising bispecific or trispecific antibodies can overcome these developmental hurdles in order to advance into human testing as novel and potentially powerful therapeutic or prophylactic agents against HIV-1. Indeed, several of these novel candidates are already in clinical development (Figs. 1 and 5). Below, we present a case study of one such bispecific antibody against HIV-1.

## Case study: quality by design approach to engineer a HIV-1 bispecific antibody with improved developability properties

As discussed earlier, 10E8.2/iMab [25] is a CrossMAb format bispecific antibody in which one antigen binding arm (iMab) targets the human CD4 receptor via the Fab of the humanized mAb ibalizumab [23], and a second antigen binding arm (10E8.2) targets the HIV-1 Env MPER via a variant of the human mAb 10E8 (Fig. 1d) [3]. The positioning of CD4- and MPER-targeting arms in this CrossMAb format produces a bispecific antibody with exquisitely potent and broad HIV-1 antiviral activity, neutralizing 100% of circulating HIV-1 strains in a 118 multi-clade panel with an IC_50_ geometric mean of 0.002 μg/mL, > 97% of this panel with an IC_80_ geometric mean of 0.006 μg/mL, and > 98% of a second 200 virus Clade C panel with similar antiviral potencies [25]. 10E8.2/iMab also potently inhibited HIV-1 in vivo, reducing viral load in HIV-1-infected humanized mice by 1.7 log_10_ and providing 100% protection against systemic challenge with a tier-2 R5 virus [25].

Despite this impressive antiviral activity in vitro and in vivo, a short-term “stress test” of 10E8.2/iMab revealed that this bispecific antibody starts to precipitate soon after incubation at 50 °C, suggesting a potential thermoinstability and aggregation propensity of this molecule under certain conditions. Five different CrossMAb format bispecific antibodies are currently in the clinic [72–76], indicating that the CrossMAb technology itself is not the cause of this thermoinstability and aggregation propensity. Additionally, other iMab-based CrossMAbs and the ibalizumab mAb did not exhibit such a high level of thermoinstability, indicating that this arm of 10E8.2/iMab was likely not causing this issue. However, the parental mAb 10E8 was previously reported to have poor solubility and a tendency to precipitate [77], suggesting that the MPER-binding arm in 10E8.2/iMab was most likely responsible for the insolubility observed at high temperatures. This inherent biophysical property had the potential to limit the further development of this potent bispecific antibody.

Hydrophobic residues constantly or dynamically exposed on the surface of proteins often result in aggregation as protein concentration increases [31, 78]. Therefore, a quality by design (QbD) approach was taken to identify and systematically mutate externally-facing hydrophobic residues on the 10E8.2 arm of 10E8.2/iMab and to replace them with hydrophilic residues in an effort to find a functional variant with improved solubility. Out of 17 antibody variants engineered, hydrophobic to hydrophilic mutations at 6 residues in 10E8.2/iMab retained satisfactory functional activity, and combinations of these 6 mutations were subjected to biophysical characterizations to determine if there was any improvement in solubility.

**Fig. 2:**
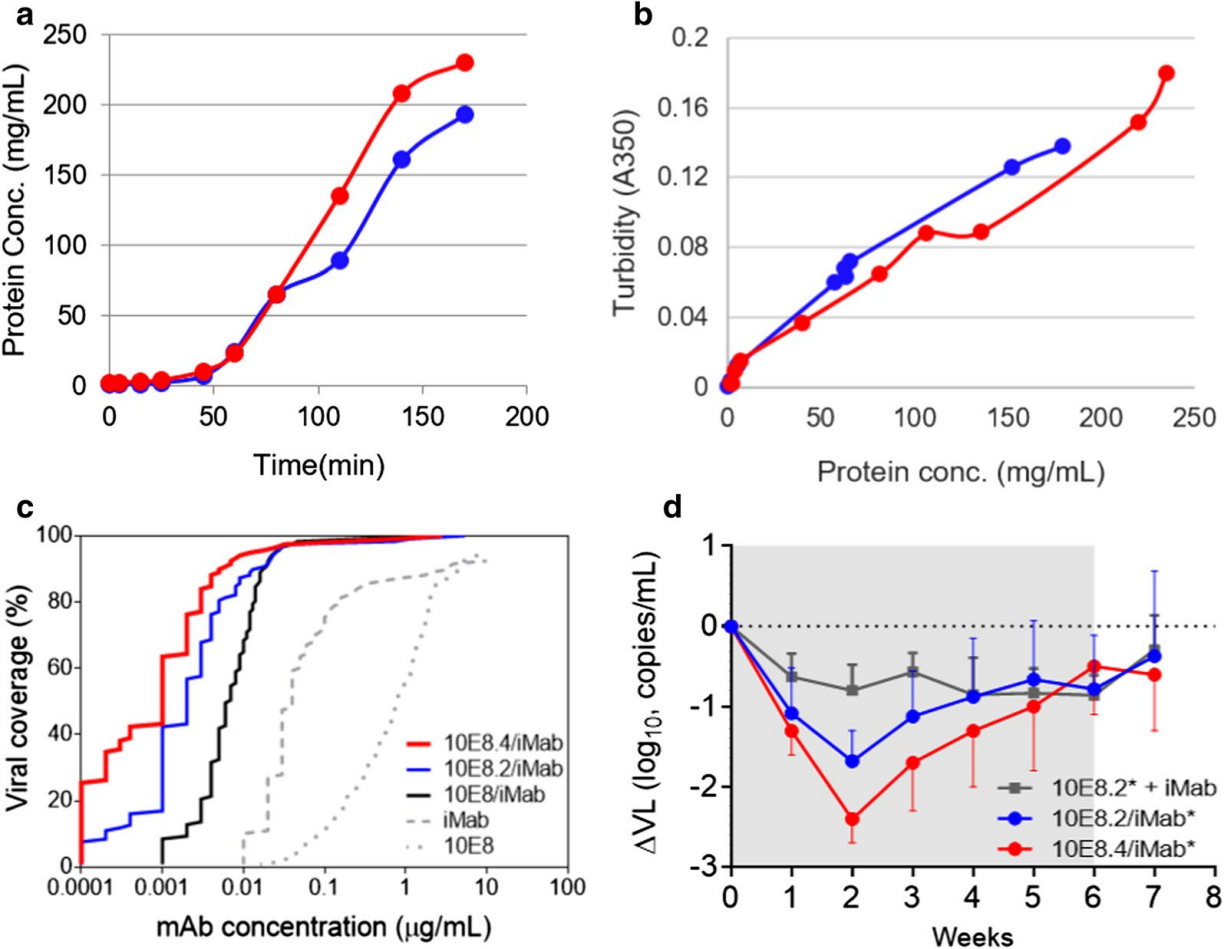
Improved solubility and antiviral activity of 10E8.4/iMab. **a** Apparent solubility and **b** turbidity of 10E8.4/iMab and its predecessor variant 10E8.2/iMab. **c** Percent of a 118 Tier-2 HIV-1 Env pseudovirus panel neutralized (based on IC_50_ values) by 10E8.4/iMab and its predecessor variants 10E8/iMab and 10E8.2/iMab. Parental mAbs iMab and 10E8 are included for reference. **d** Decrease in viral load by 10E8.4/iMab and its predecessor variant, 10E8.2/iMab, in HIV-1-infected humanized mice. Shaded area indicates the period of weekly antibody administration. Error bars = SD. * = N297A mutant variant of each bispecific antibody. As reported previously [84], this mutation in the Fc region of each bispecific antibody is required for evaluation of non-FcR binding human antibodies in the murine model

The apparent solubility of 10E8.4/iMab was determined in comparison to 10E8.2/iMab by formulating both antibodies at identical starting concentrations and subjecting them to ultracentrifugation. At concentrations above 50 mg/mL, 10E8.4/iMab showed consistently higher protein concentrations and solubility over time as compared to 10E8.2/iMab, and the apparent solubility, or maximum concentration achieved, of 10E8.4/iMab was calculated to be > 230 mg/mL (Fig. 2a). This improvement in solubility, combined with long-term stability data, strongly suggests that 10E8.4/iMab could be formulated not just for intravenous administration to humans, but also at the higher concentrations required for subcutaneous administration since volume constraints are often a concern for delivery by this latter route. Consequently, 10E8.4/iMab delivery by both of these routes of administration will be evaluated clinically.

The turbidity of 10E8.2/iMab and 10E8.4/iMab at various protein concentrations was also evaluated in order to draw a correlation between these two parameters. While the turbidity of both 10E8.2/iMab and 10E8.4/iMab expectedly increased with protein concentration over time, 10E8.2/iMab showed consistently higher turbidity than 10E8.4/iMab at the same protein concentrations over 100 mg/mL, indicating improved solubility of 10E8.4/iMab (Fig. 2b). 10E8.2/iMab and 10E8.4/iMab were also subjected to a forced degradation analysis to determine their relative protein stabilities under thermal stress-inducing conditions. In addition to an improvement in appearance and decrease in turbidity, 10E8.4/iMab also exhibited better intact molecule purity over time by capillary electrophoresis (CE) SDS-PAGE and fewer aggregation-associated high molecular weight species over time by size exclusion chromatography, indicating its relatively better stability under thermal stress-inducing conditions as compared to 10E8.2/iMab.

In addition to its improved solubility and thermostability, 10E8.4/iMab also exhibited a 2.5-fold enhancement in antiviral potency when tested against the same panel of 118 Tier-2 HIV-1 pseudotyped viruses representing diverse clades and geographic origins described earlier (Fig. 2c). In a humanized mouse model of HIV-1 infection, weekly administrations of 10E8.4/iMab reduced the viral load of HIV-1-infected mice by 2.4 log_10_ while a maximum mean viral load reduction of ~ 1.7 log_10_ was observed in mice treated with 10E8.2/iMab (Fig. 2d).

In summary, in silico analysis of the 10E8.2/iMab sequence and structure for potential aggregation-inducing hotspots revealed a number of residues that could be detrimental for the developability of this potent bispecific antibody for the clinic. A potential setback as a result of these inherent molecular properties may often not be realized until significant funds and time are exerted for the advancement of a particular therapeutic into the clinic. However, utilizing a QbD approach to systematically mutate each of these hotspot residues individually, and iteratively testing combinations of these engineered variants for improved product quality attributes, led to the identification of a new improved variant, 10E8.4/iMab. While there is always the theoretical risk that engineering new residues into an antibody may result in unanticipated immunogenicity, the likelihood of this is uncertain and cannot be definitively assessed until clinical investigation [71]. Therefore, based on its superior solubility and stability and its further improved potent in vitro and vivo antiviral activity, 10E8.4/iMab was selected as a clinical lead candidate for further development.

## Case study: cell line development of a CrossMAb format HIV-1 bispecific antibody

Cell line development in preparation for reproducible production of a given mAb therapeutic for human use is now an established process, as evidenced by the > 85 mAbs approved for commercial use by the US FDA for the treatment of a number of different human diseases [79], and this does not include the many more mAbs that are currently in preclinical and clinical development. The heavy chain and light chain of a given mAb are encoded together on one plasmid that contains an antibiotic resistance selection marker or separately on two plasmids, each with its own unique antibiotic resistance selection marker. These plasmids are then stably transfected into a cell line. After transfection, single clones that produce high titers of the mAb, as determined by Protein A binding to the Fc region of antibody secreted into the supernatant, are selected and further characterized in order to downselect a lead clone for GMP master cell bank production. For the cell line development of 10E8.4/iMab, a modified approach was necessary due to a total of four separate open reading frames (encoding 10E8.4 heavy chain, 10E8.4 light chain, iMab heavy chain, and iMab light chain) that need to be stably transfected. By transient transfection, encoding four different open reading frames in four separate plasmids reproducibly produces CrossMAb bispecific antibodies with > 80% intact molecule purity [46]. For stable transfection, however, encoding these four different open reading frames in four separate plasmids was not feasible because the high level of antibiotic selection pressure against four distinct markers would drastically reduce the number of surviving clones that could be screened for high titer-producing antibody levels.

**Fig. 3:**
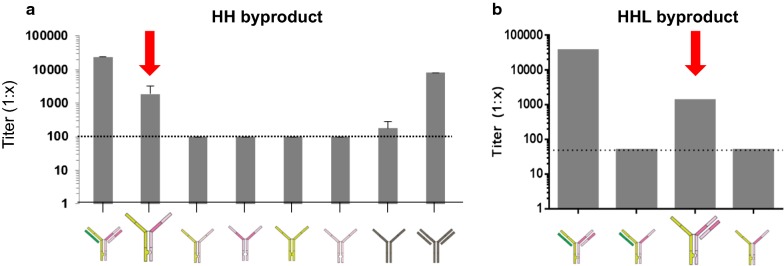
Secretion of CrossMAb byproducts that could hinder cell line development and clone selection. Detection of the indicated antibody or antibody byproduct in supernatant after transient transfection of ORFs encoding for the antibody chains indicated in the schematics. Protein detection in supernatant was determined by Protein A binding ELISA. Dashed lines indicate the assay limit of detection. Error bars = SD. **a** HH dimer byproducts and **b** HHL impurity byproducts were readily detected in supernatants

**Fig. 4:**
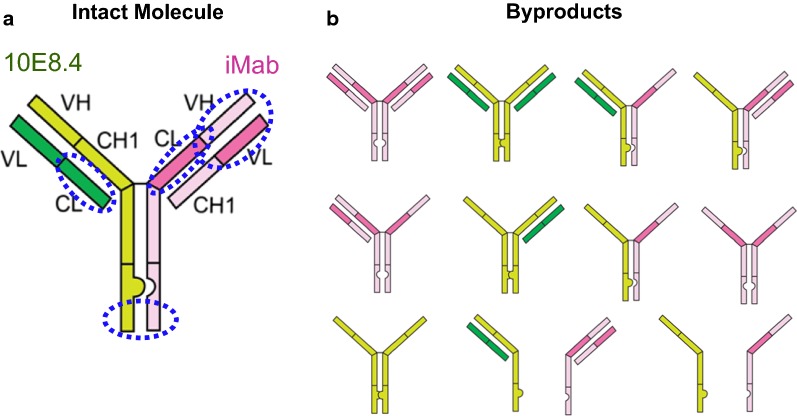
CrossMab format for bispecific antibody production. Knob-in hole mutations in the CH3 domains favor heterodimer heavy chain formation. CH1-CL crossover in one arm of the CrossMAb favors proper light chain association with its cognate heavy chain. In combination, **a** intact molecule production and secretion is favored and **b** byproduct production and secretion is disfavored. Dashed blue circles indicate target domains that, when detected simultaneously, ensure a greater percentage of intact molecule

After attempting stable transfection of 10E8.4/iMab encoded in two or three plasmid configurations, and screening for high titer clones by Protein A binding to the Fc region of the secreted antibody, the highest level of intact molecule purity produced from a stable pool of clones was 68.5%, which is too low to support a viable upstream production and downstream purification strategy for clinical development. Analysis by non-reduced CE SDS-PAGE of the impurities present in the supernatant of the top stable pools revealed a significant fraction of heavy chain–heavy chain (HH) and heavy chain–heavy chain–light chain (HHL) impurities present in the clonal supernatant. Theoretically, the knob-in-hole and light chain crossover technologies incorporated into the CrossMAb format should prevent these impurities from being secreted. However, our investigational analyses revealed that, if all four ORFs are not present in the transfection mix, impure byproducts can be readily secreted. For example, transfection of 10E8.4 HC and iMab HC, without their cognate light chains, can be secreted (Fig. 3a), as can 10E8.4 HC, iMab HC and iMab LC impurities (Fig. 3b). Fundamental biological studies of monoclonal antibody secretion indicate that antibody HCs are not typically secreted from cells without their cognate LCs associated, and a closer investigation revealed that the signal for this antibody secretion is associated with close proximity of the CH1 domain of a nascently formed antibody HC with the CL domain of a nascently formed antibody LC in the endoplasmic reticulum [80]. Due to the unique configuration of the light chain crossover technology in CrossMAb antibodies, however, the CL of ibalizumab is located on the “heavy chain” (Fig. 4a), and we speculate that the close proximity of this CL in the ibalizumab “heavy chain” and the CH1 domain in the 10E8.4 HC can trigger antibody secretion without their cognate LCs. With consideration to our stable cell line transfection efforts, one can easily envision how overexpression or underexpression of one or more of the four bispecific antibody chains in a stable cell line could allow for permissive secretion of HH or HHL impurities if the missing chain(s) is produced at relatively low levels. Also, since our initial screening strategy, which is commonly used for mAb cell line selection, indiscriminately selected for high-producer clones by Fc-binding to Protein A, it was impossible to differentiate clones producing the intact HHLL molecule from those that produced HHLL along with a mixture of HH and HHL impurities since all of these products would have nearly identical binding properties to Protein A.

To overcome these challenges, we undertook a multi-pronged approach that specifically addressed the unique differences in cell line development between a typical mAb and the 10E8.4/iMab bispecific CrossMAb. We generated new two and three plasmid vector combinations encoding the 10E8.4 HC, 10E8.4 LC, iMab HC and iMab LC in several different permutations, and transiently transfected them at numerous ratios to identify the plasmid combinations and ratios that could give the best percentage of intact molecule purity by transient transfection in order to downselect the most promising set of plasmids and conditions to advance into stable transfection studies. In all, more than 20 different plasmid configurations and conditions were evaluated. Next, by designing a new screening strategy that recognized four distinct domains of 10E8.4/iMab simultaneously rather than only its singular Fc region, we could select for high titer producing clones with better assurance that they were producing fully intact HHLL molecules rather than byproduct impurities (Fig. 4a). In effect, if we equate identifying a high titer producing clone within a large pool of stably transfected clones to identifying a needle in a haystack, our redesigned screening strategy was a powerfully tuned magnet that could sift through the “hay” of clones to find our high titer producing “needle.” To do this, we developed new FRET-based methods to simultaneously detect multiple distinct arms within the 10E8.4/iMab intact molecule, and utilized CE SDS-PAGE as our analytical screening tool to confirm intact molecule purity levels relative to byproduct impurities. If a suitable bispecific ELISA-based method was available that could simultaneously detect both functional antibody arms, this could also be employed. Finally, we plated and screened over five times as many clones as was done for a typical mAb cell line development program in order to ensure that we could identify a suitable lead clone. In effect, now equipped with our powerful screening strategy and magnet, we could increase the size of the haystack in order to ensure that one or more of our needles was contained within it. These laborious efforts proved fruitful, and a final lead cell line clone was identified that produced 10E8.4/iMab at > 90% intact molecule purity after a simple 1-step purification and at a titer of > 3 g/L. This titer is on par with excellent mAb-producing clones and much better than what is expected for a typical bispecific antibody. Additional polishing steps purified 10E8.4/iMab to > 97%, which is well within the range of purity acceptable to advance this novel and potent HIV-1 bispecific antibody into clinical evaluation.

## Conclusions

**Fig. 5:**
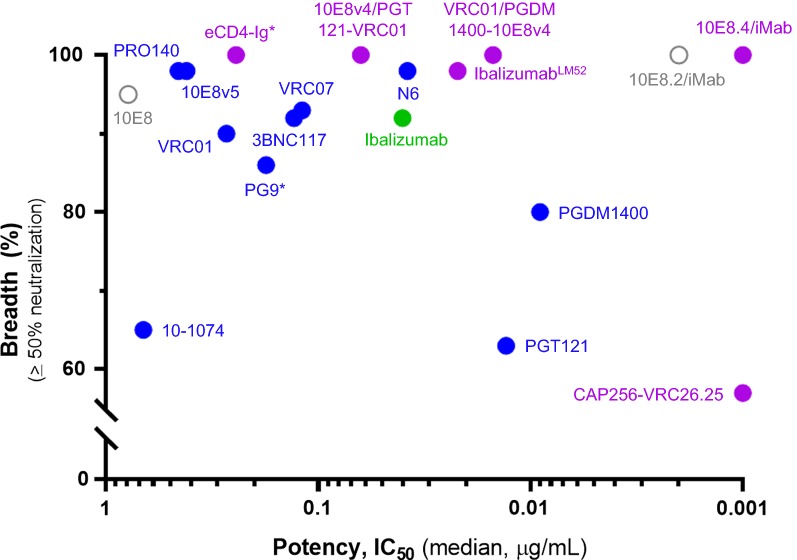
Antiviral potency and breadth of HIV-1 mAbs and multi-specific Abs. HIV-1 mAbs and multi-specific antibodies that are licensed (green), in clinical trials (blue), or in clinical development (purple). Open circles represent earlier variants of antibodies in development that are presented. *Means antibodies were delivered by AAV. Figure adapted from Xu et al., 2017 and additional published reports [54, 85, 86]

The new generation of broadly neutralizing mAbs against HIV-1 has given the field a new avenue of hope for prophylactic and therapeutic possibilities to reduce the existing HIV-1 burden. In addition to the recent FDA approval of ibalizumab (Trogarzo^®^) for use as salvage therapy in patients whose viruses are resistant to multiple existing antiretroviral drugs, VRC01 is currently in two Phase 2b efficacy trials for HIV-1 prevention in HIV-1 uninfected men and transgender persons who have sex with men in the United States, Peru, Brazil, and Switzerland (HVTN 704/HPTN 085) and in HIV-1 uninfected sexually active women in seven countries in sub-Saharan Africa (HVTN 703/HPTN 081) [26, 81]. Known as the Antibody Mediated Prevention (AMP) Studies, the lessons learned from these VRC01 Phase 2b efficacy trials will be of tremendous benefit the field of antibody-mediated HIV-1 prevention. It is clear, however, that drastic improvements to antibody potency and breadth will be required in order to produce a feasible antibody regimen which could be used widespread and which could limit the emergence of viral resistance well known to those in the HIV-1 treatment field. Bispecific and trispecific antibodies offer a new beacon of hope to combat viral resistance by improving neutralization breadth and, in some cases, by drastically improving antiviral potency by orders of magnitude over the best HIV-1 mAbs currently in existence (Fig. 5). However, the development of these HIV-1 multi-specific antibodies is not without its own challenges. The potential for aggregation, immunogenicity and low GMP cell line titers is an issue for any antibody, and these are amplified in cases of multi-specific antibodies due to their unique formats and engineered properties required to create their multi-specificity. In addition to the challenges discussed in this review, other downstream chemistry, manufacturing and controls obstacles such as antibody purification and stability of engineered multi-specific molecules may exist. Further in development, nonclinical challenges, such as manufacturing and incorporating parental mAb control groups into GLP toxicology programs in the event that safety signals for a given multi-specific antibody requires further investigation, may also arise [82]. During clinical investigation, pharmacokinetic and anti-drug antibody assays must be able to detect each specificity within a given multi-specific antibody, and therefore reagents or assays that can detect each unique epitope within a given HIV-1 multi-specific antibody are preferred [83].

Despite these challenges, the tremendous opportunities for bispecific and trispecific antibodies against HIV-1 are readily evident. Applying the same creativity and rigor to the development and manufacture of HIV-1 multi-specific antibodies as that which was used for their creation and initial characterization promises to offer to the field a new generation of potent and broad multi-specific antibodies that could be ready to enter the clinic within the same timeframe as a typical mAb. In parallel, the ongoing discovery of ever more potent and broadly neutralizing HIV-1 mAbs continues to provide new and improved foundational starting blocks for incorporation into multi-specific antibodies. How we create and advance these powerful multi-specific antibodies for the prevention and treatment of HIV-1 will only be limited by our imagination, rigor and diligence.

## Abbreviations

AAV: adeno-associated virus
bp: basepairs
CE: capillary electrophoresis
CODV-Ig: cross-over dual variable immunoglobulin G
DNA: deoxyribonucleic acid
ds: double-stranded
Env: envelope
HH: heavy chain–heavy chain
HHL: heavy chain–heavy chain–light chain
IC: inhibitory concentration
HIV-1: human immunodeficiency virus 1
KiH: knob-in hole
iMab: ibalizumab
mAb: monoclonal antibody
MPER: membrane proximal external region
P140: PRO140
QbD: quality by design
SHIV: simian human immunodeficiency virus
scFv: single-chain variable fragment
